# Treatment with vascular endothelial growth factor-A worsens cognitive recovery in a rat model of mild traumatic brain injury

**DOI:** 10.3389/fnmol.2022.937350

**Published:** 2022-10-26

**Authors:** Mujun Sun, Tamara L. Baker, Campbell T. Wilson, Rhys D. Brady, Richelle Mychasiuk, Glenn R. Yamakawa, Anh Vo, Trevor Wilson, Stuart J. McDonald, Sandy R. Shultz

**Affiliations:** ^1^Department of Neuroscience, Central Clinical School, Monash University, Melbourne, VIC, Australia; ^2^Monash Health Translation Precinct, Monash University, Melbourne, VIC, Australia; ^3^Department of Medicine, The University of Melbourne, Parkville, VIC, Australia; ^4^Health and Human Services, Vancouver Island University, Nanaimo, BC, Canada

**Keywords:** concussion, cerebrovascular, VEGF-A, intracerebroventricular administration, neuroinflammation, hypoxia

## Abstract

Mild traumatic brain injury (mTBI) is a common and unmet clinical issue, with limited treatments available to improve recovery. The cerebrovascular system is vital to provide oxygen and nutrition to the brain, and a growing body of research indicates that cerebrovascular injury contributes to mTBI symptomatology. Vascular endothelial growth factor-A (VEGF-A) is a potent promoter of angiogenesis and an important modulator of vascular health. While indirect evidence suggests that increased bioavailability of VEGF-A may be beneficial after mTBI, the direct therapeutic effects of VEGF-A in this context remains unknown. This study therefore aimed to determine whether intracerebroventricular administration of recombinant VEGF-A could improve recovery from mTBI in a rat model. Male and female Sprague–Dawley rats were assigned to four groups: sham + vehicle (VEH), sham + VEGF-A, mTBI + VEH, mTBI + VEGF-A. The mTBI was induced using the lateral impact model, and treatment began at the time of the injury and continued until the end of the study. Rats underwent behavioral testing between days 1 and 10 post-injury, and were euthanized on day 11 for post-mortem analysis. In males, the mTBI + VEGF-A group had significantly worse cognitive recovery in the water maze than all other groups. In females, the VEGF treatment worsened cognitive performance in the water maze regardless of mTBI or sham injury. Analysis of hippocampal tissue found that these cognitive deficits occurred in the presence of gene expression changes related to neuroinflammation and hypoxia in both male and female rats. These findings indicate that the VEGF-A treatment paradigm tested in this study failed to improve mTBI outcomes in either male or female rats.

## Introduction

Mild traumatic brain injury (mTBI), often referred to as concussion, has an estimated annual incidence of 64–74 million worldwide (Dewan et al., 2018). The majority of mTBI patients experience debilitating short-term symptoms, and recent evidence indicates that >30% of mTBI patients will experience persisting post-concussion symptoms (PPCS) that last for months to years (Voormolen et al., 2019, 2020; Machamer et al., 2022). Despite the increasing awareness of the socioeconomic costs and poor outcomes following mTBI, the clinical management of these injuries remains difficult with limited evidence-based intervention options available (Silverberg et al., 2020).

The kinetic forces at the time of mTBI, as well as the secondary injury cascades that follow, can result in deformation, dysfunction, and injury to the cerebrovascular system that may manifest in cerebral blood flow changes, perivascular neuroinflammation, blood brain barrier (BBB) damage, and microhemorrhage (Bonne et al., 2003; Marchi et al., 2013; Wang et al., 2014; Long et al., 2015; Tagge et al., 2018; Baker et al., 2021). Notably, evidence from both animal and human studies indicate that cerebrovascular injury is associated with mTBI symptoms and functional deficits (Bonne et al., 2003; Wang et al., 2014; Huang et al., 2015; Long et al., 2015). Furthermore, aerobic exercise, one of the few promising mTBI interventions currently available to patients (Leddy et al., 2019), is known to modify several important mediators of cerebrovascular health – including the upregulation of vascular endothelial growth factor A (VEGF-A; Morland et al., 2017; Zarezadehmehrizi et al., 2021).

VEGF-A is a potent promoter of angiogenesis (i.e., the growth of new blood vessels) and modulator of vascular health (Zeng et al., 2001; Ferrara, 2004). Treatment with, or upregulation of, VEGF-A has been found to be neuroprotective in animal studies of severe TBI, promoting microvascular recovery, neurogenesis, and functional improvements (Lee and Agoston, 2010; Siddiq et al., 2012). On the other hand, treatment with bevacizumab, a VEGF-A neutralizing antibody, resulted in worse brain damage and neurological deficits after experimental severe TBI (Tado et al., 2014). This evidence, coupled with findings that aerobic exercise (i.e., an intervention that upregulates VEGF-A) can facilitate mTBI recovery, warrants further investigation into the therapeutic benefits of VEGF-A in mTBI. As such, the present study investigated whether intracerebroventricular administration of recombinant VEGF-A would improve mTBI recovery in a rat model. Both male and female rats were included because past findings suggest that mTBI outcomes, including cerebrovascular measures, may differ between the sexes (Wright et al., 2017, 2021; Jullienne et al., 2018; Hamer et al., 2020; Mcdonald et al., 2021).

## Materials and methods

### Animals

Sprague–Dawley rats (42 males and 42 females) were obtained from the Monash Animal Research Platform. Rats were housed under a 12:12 light/dark cycle, with access to food and water *ad libitum*. Rats were group housed until cannula implant, at which point they were single housed for the remainder of the study. The rats were 10 weeks old at the time of mTBI/sham injury. All procedures performed on the rats were approved by the Monash University Animal Ethics Committee and were conducted in fulfilment of the requirements of the Australian Code of Practice for the Care and Use of Animals for Scientific purposes.

### Experimental groups and study design

**Figure 1:**
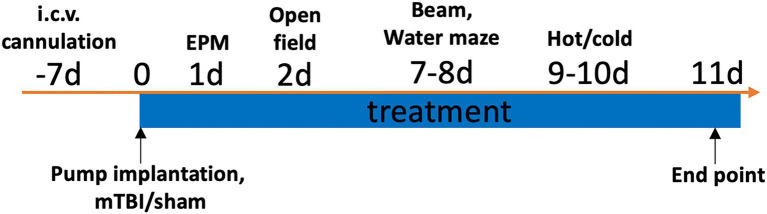
Study timeline. One week after the ICV cannulation, rats were implanted with an osmotic pump and immediately received either a LI induced mTBI or sham injury. Rats received the VEGF-A or VEH treatment throughout the study. Behavioral testing was conducted over the subsequent 10 days, and post-mortem analysis was conducted at 11 days after the injuries.

All rats were implanted with a cannula in the lateral ventricle of the brain. One week post implantation, rats received either a lateral impact (LI) induced mTBI or sham injury, and were randomly assigned to receive either the recombinant rat VEGF-A (Sigma V3638) or vehicle (VEH) treatment (artificial cerebrospinal fluid, ACSF; *In Vitro* Technologies) (Figure 1). As such, there were four experimental groups: Sham+VEH (*n* = 10/sex), Sham+VEGF (*n* = 10/sex), LI + VEH (*n* = 11/sex), LI + VEGF (*n* = 11/sex). Behavioral testing was conducted over the subsequent 10 days, and rats were euthanized 11 days after the mTBI for tissue collection and post-mortem analysis (Figure 1).

### Intracerebroventricular cannulation and drug administration

One week prior to mTBI, rats underwent surgery to implant an ICV cannula (Alzet®, brain kit 2). The rat was placed in an induction box and anesthesia was induced by 4% isoflurane. The anesthesia was maintained by 2% isoflurane *via* a nose cover throughout surgery. The cannula was implanted into the lateral ventricle at the following coordinates relative to the bregma: posterior 0.2 mm, lateral 1.6 mm and at 4.5 mm depth from skull surface (Paxinos and Watson, 2006; Shultz et al., 2008; Lee and Agoston, 2010). The cannula was attached to the skull using dental cement and stabilized by the addition of two stainless-steel screws inserted into the skull surrounding the cannula. The cannula tubing was sealed, and the rat’s head was sutured. All rats received a dose of Buprenorphine (0.05 mg/kg) during the surgery, and again at 12-and 24-h post-surgery.

One week after ICV cannulation, a mini osmotic pump (Alzet®, model 2002) was implanted subcutaneously between scapulae under anesthetic condition and connected to the cannula tubing (Shultz et al., 2015). Bovine serum albumin (BSA; Sigma A4919) was added at 1 mg/ml as carrier proteins (Lee and Agoston, 2010). The osmotic pump then delivered either artificial cerebrospinal fluid (ACSF; with BSA) or VEGF-A (10 μg/ml in ACSF with BSA) to the brain ventricle at a rate of 0.5 μl per hour. This treatment protocol was based on previous studies (Sun et al., 2003; Lee and Agoston, 2010).

### The LI model of mTBI

Immediately after the osmotic pump implantation, the rat underwent the LI/sham. As previously described (Mychasiuk et al., 2016a; Wright et al., 2017), rats were placed sternally on the device on a Teflon® board. The impactor was set to propel a 50 mg weight using pneumatic pressure at 14 m/s (~137Gs) into a helmet-like metal plate (30 × 13 × 3 mm) which was resting against the lateral side of the skull. This impact resulted in the acceleration of the rat head and 180-degree rotation of the rat body. The time taken for the rat’s self-right reflex to occur was recorded to confirm the mTBI (Table 1). The sham procedure was identical to the LI, except that the impact was not induced.

**Table 1:** Acute injury measures.

	Sham+VEH	Sham+VEGF	LI + VEH	LI + VEGF
Male	184.4 ± 23.55	221.8 ± 31.22	303.8 ± 39.99*	268.1 ± 41.15*
Female	159.3 ± 19.9	187.1 ± 28.35	315 ± 50.84*	296 ± 33.01*

For each sex, the LI groups had significantly self-righting reflex times (seconds ± SEM) than the SHAM groups. For each sex, there were no statistically significant differences between the two LI groups (*p* > 0.05).

* For each sex, LI groups different than SHAM groups, *p* < 0.05.

### Behavioral testing

#### Elevated plus maze

One day after injuries, rats underwent testing in an EPM to measure anxiety-like behavior (Shultz et al., 2012; Sun et al., 2019). The maze is a cross shaped apparatus consisting of 4 arms (two opened arms and two closed arms, each 50 cm x 10 cm) extending out from a central platform (10 cm x 10 cm). The enclosed arms were shielded by walls 30 cm high. To begin the test, the rat was placed in the centre platform facing an open arm and allowed to explore the maze freely for 5 min. The rat’s movement was tracked by the tracking software TopScan™ 3.0 (Clever Sys., Inc., United States). A percentage score was calculated as time in the open arms/ (time in the open arms + time in the closed arms) to analyze anxiety-related behavior (Shultz et al., 2011, 2012; Pham et al., 2021). The closed arm entrance frequency was calculated to assess gross motor function and locomotion.

#### Open field

Two days after injuries, the rats underwent open field testing to analyze locomotion (Shultz et al., 2014; Sun et al., 2019). This maze consisted of a circular open arena (100 cm diameter) surrounded by 30 cm high walls. A circular area (66 cm diameter) in the arena was defined as the middle field. The rat was placed into the centre of the arena and allowed to explore for 5 min. The rats were tracked using TopScan™ 3.0 software which measured time spent in the middle field, as well as total distance travelled in the whole open field.

#### Morris water maze

One week after the mTBI, rats underwent water maze testing to assess cognitive function (Bao et al., 2012; Shultz et al., 2012, 2013; Sun et al., 2019). The water maze consisted of a large circular pool (174 cm in diameter) filled with warm water (26–29°C). Submerged 3.5 cm below the surface of the water in one quadrant of the pool was a small hidden platform (10 × 10 cm) which allowed the rats to escape the water. Four different and visible cues were fixed around the perimeter of pool for spatial orientation. Rats completed 10 trials each day, over 2 days. On the first day (acquisition session), the platform was placed in between the south and east quadrants. On day two (reversal session) the platform was moved to between the north and west quadrants. Each trial began at one of four randomized start locations around the perimeter of the pool (i.e., north, south, east, and west), where the rat was released from the starting location facing the wall. All rats were given a maximum of 60 s to explore the arena and locate the platform. If the rat did not locate the platform by the time limit, they were led to the platform by the researcher. After 15 s on the platform, the rat was removed from the pool and placed under a heated lamp. The rat’s movement was tracked by the tracking software TopScan™ 3.0 (Clever Sys., Inc., United States). Search time to reach the platform and the number of direct and circle swims were calculated as measures of spatial cognition.

#### Beam

One week after the injuries, the beam task assessed each rat on its sensorimotor function through its ability to traverse a beam 100 cm long and 2 cm in width (Shultz et al., 2013; Sun et al., 2019). Rats were first habituated to the task the day prior to testing by completing the task five times on a beam 4 cm in width and five times on the 2 cm wide beam. Beam testing involved 10 trials on the 100 cm long × 2 cm wide beam. The time taken for each rat to complete the beam was measured, as well as the number of slips and falls. A maximum time of 60 s was allowed for the rat to cross the beam. Slower times to cross the beam and an increased number of slips were suggestive of a loss of function in both locomotion and balance (Shultz et al., 2013). Slips were defined as any limb that slipped from the beam.

#### Hot/cold plate

The hot/cold plate was utilized to test thermal hyperalgesia/allodynia. Hot and cold plate test sessions were conducted at 10 days post-injury. The apparatus is comprised of a circular plate 19 cm in diameter enclosed by a clear acrylic cylinder 25 cm tall and covered by a lid. Rats initially underwent a habitation session per day for 2 days before the testing and the test sessions were conducted on the 3^rd^ day. During habituation, each rat was placed in the cylinder at room temperature for 2 min. During the test, each rat was placed in the cylinder at 52°C and observed until the rat displayed nociceptive behavior, such as forepaw or hindpaw withdrawal or licking, stamping of the feet, or jumping, at which point the rat was removed from the plate and returned to its cage. The response latency (seconds) was then recorded (Salberg et al., 2020). After an hour, the rats were again placed on the plate, this time cooled to 2°C and were observed for nociceptive behavior. Again, the response latency was recorded. A reduced response latency suggested increased susceptibility to pain in the rats (Le Bars et al., 2001).

### Gene expression analysis

At 11 days post injury, rats were anesthetized and decapitated. The ipsilateral hippocampus was dissected, rapidly frozen, and stored at −80°C. Gene expression analysis was used to examine genes related to vascular health, neuroinflammation, and hypoxia. Total RNA was isolated from 20 mg tissue by a RNeasy® Mini Kit (Qiagen) running by QIAcube (Qiagen). 200 ng yielded RNA were proceeded to cDNA synthesis using Quantabio qScript XLT cDNA SuperMix (Quantabio). Multiplex qPCR was performed with Fluidigm BioMark HD™. For each sample, 1.25 μl of the resulting cDNA were combined with 3.75 μl of Sample Pre Mix (Life Technologies TaqMan® PreAmp Master Mix and Pooled Taqman assays) and preamplified for 14 cycles. The reaction products were diluted 1: 5, and loaded onto the Gene expression IFC according to Fluidigm® IFC Standard Taqman Gene expression workflow. 42 TaqMan® gene expression assays related to neuroinflammation, hypoxia injury and vascular health, and 4 housekeep gene assays were used and detailed in Table 2. Cycle threshold (Ct) values were collected for analysis, using the 2^−ΔΔCt^ method.

**Table 2:** The TaqMan^®^ gene expression assays.

Gene	Thermo fisher assay
*Vascular health*	
*VEGF A*	Rn01511602_m1
*MMP9*	Rn00579162_m1
*MMP2*	Rn01538169_m1
*S1P1*	Rn02758712_s1
*VEGF R2*	Rn00564986_m1
*AQP4*	Rn01401322_m1
*TGFB1*	Rn00572010_m1
*Neuroinflammation*	
*TMEM119*	Rn01480631_m1
*MCSF*	Rn01522726_m1
*IL6*	Rn01410330_m1
*TNF*	Rn99999017_m1
*IL1α*	Rn00566700_m1
*IL1β*	Rn00580432_m1
*IFNG*	Rn99999014_m1
*MCP1*	Rn00580555_m1
*CCL5*	Rn00579590_m1
*CXCR4*	Rn01483207_m1
*IBA1*	Rn00567906_g1
*GFAP*	Rn00566603_m1
*CD68*	Rn01495634_g1
*CD86*	Rn00571654_m1
*CCR5*	Rn00588629_m1
*CCR2*	Rn01637698_s1
*NLRP3*	Rn04244622_m1
*Hypoxia*	
*HIF1α*	Rn01642006_m1
*HIF2α*	Rn00576515_m1
*HIF1β*	Rn00688999_m1
*FIH1*	Rn01766292_m1
*EPO*	Rn01481376_m1
*iNOS*	Rn00561646_m1
*nNOS*	Rn00583793_m1
*eNOS*	Rn07312037_g1
*CFOS*	Rn02396759_m1
*FIGF*	Rn00582193_m1
*GLUT1*	Rn01417099_m1
*Cell stress*	
*HSP90AB1*	Rn01511686_g1
*HSP27*	Rn00583001_g1
*HSPA1A*	Rn04224718_u1
*HSF1*	Rn00801772_m1
*Others*	
*SDHA*	Rn00590475_m1
*CASP9*	Rn00581212_m1
*CYBB*	Rn00576710_m1
*Housekeeping genes*	
*HPRT*	Rn01527840_m1
*B2M*	Rn00560865_m1
*YWHAZ*	Rn00755072_m1
*PPIA*	Rn00690933_m1

### Statistical analysis

Data were analyzed with SPSS 23.0 software (IBM Corp, Armonk, United States). To simplify statistical analyses, male and female data were analyzed separately. Water maze search time was analyzed by mixed-design analysis of variance (ANOVA), with treatment (VEGF vs. VEH) and injury (sham vs. LI) as between-subjects variables and day as the within-subjects variable. Water maze data is presented in blocks of two trials in the figures for visual clarity. Other behavior and gene expression outcomes were analyzed by two-way ANOVA, with treatment and injury as between-subject factors. Bonferroni post-hoc comparisons were carried out when appropriate. Statistical significance was set as *p* < 0.05.

## Results

### VEGF treatment worsens cognitive deficits after mTBI in male rats

**Figure 2:**
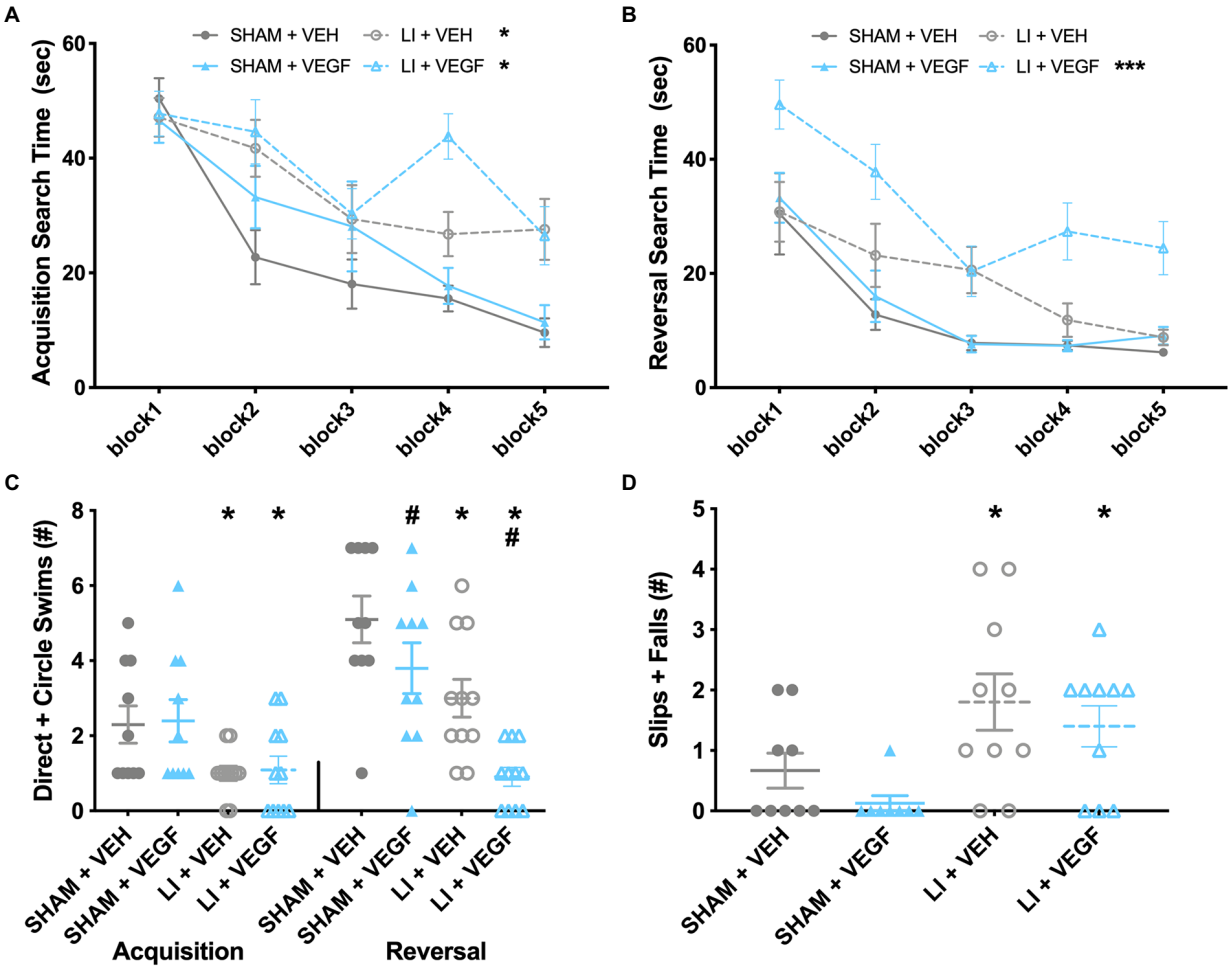
Behavior findings in male rats. **(A)** During water maze acquisition, male LI rats spent significantly longer than sham rats to find the platform on trials 3, 7, 8, 9, and 10. **(B)** During water maze reversal, LI + VEGF rats took longer to find the platform than all other groups. **(C)** LI rats had fewer direct and circle swims than sham rats during acquisition. During reversal, both VEGF and LI rats had fewer direct and circle swims. **(D)** LI rats had more slips and falls than the shams on beam task. *, LI rats significantly different than sham rats; #, VEGF treated rats significantly different than VEH treated rats; ***, different than all other groups; *p* < 0.05. Sham+VEH, *n* = 10; Sham+VEGF, *n* = 10; LI + VEH, *n* = 11; LI + VEGF, *n* = 11; except for beam, Sham+VEH, *n* = 9 Sham+VEGF, *n* = 8; LI + VEH, *n* = 10; LI + VEGF, *n* = 10. Error bar = SEM; water maze block = mean of every two trials.

During water maze acquisition, there was a significant injury by trial interaction (*F*_9,342_ = 2.138, *p* < 0.05) on the measure of search time, with male LI rats spending significantly longer than male sham rats to find the platform on trials 3, 7, 8, 9,10 (*p* < 0.05; Figure 2A). There were also significant main effects for injury (*F*_1,38_ = 24.620, *p* < 0.001; Figure 2A) and for trial (*F*_9,342_ = 13.857, *p* < 0.001; Figure 2A) on the measure of search time. On the measure of direct and circle swims, there was a main effect of injury (*F*_1,38_ = 9.761, *p* < 0.01; Figure 2C) with male LI rats having fewer direct and circle swims than male sham rats.

During water maze reversal, there was a significant injury by treatment interaction (*F*_1,38_ = 6.498, *p* < 0.05) on the measure of search time. Post-hoc analysis found that LI + VEGF male rats took longer to find the platform than all other male groups (*p* < 0.05; Figure 2B). There were also main effects found for trial (*F*_9,342_ = 18.425, *p* < 0.001), injury (*F*_1,38_ = 28.571, *p* < 0.001), and treatment (*F*_1,38_ = 11.166, *p* < 0.01) on search time (Figure 2B). On the measure of direct and circle swims, there were main effects treatment (*F*_1,38_ = 10.272, *p* < 0.01) and injury (*F*_1,38_ = 22.252, *p* < 0.001), with both VEGF-treated and LI rats having fewer direct and circle swims than their VEH-treated and sham-injured counterparts (Figure 2C). There were no significant findings for swim speed for both acquisition and reversal, suggesting that all groups had similar swimming ability (data not shown, *p* > 0.05).

On the beam task, there was a main effect of injury (*F*_1,33_ = 11.902, *p* < 0.01; Figure 2D) indicating that the male LI rats had more slips and falls than the male shams.

No significant findings were found in the EPM, open field, or hot/cold plate for the male rats (data not shown, *p* > 0.05).

### VEGF treatment decreases anxiety-like behavior and cognitive performance in female rats

**Figure 3:**
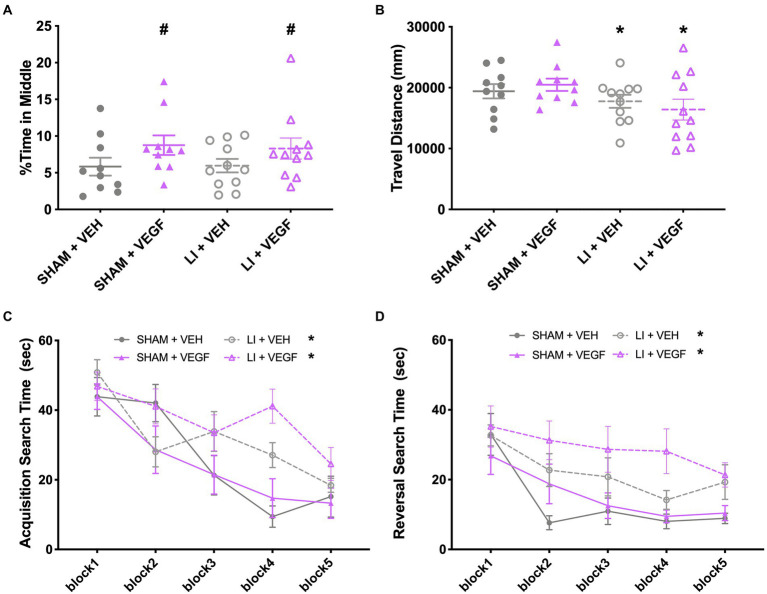
Behavior findings in female rats. In the open field, female rats treated with VEGF spent more time in the middle than VEH rats **(A)**, and female LI rats travelled less distance compared to shams **(B)**. **(C)** During water maze acquisition, LI rats took longer than the shams to find the platform on trials 6–8. **(D)** During water maze reversal, VEGF rats took longer to find the platform on trials 4 and 8 than VEH rats, and LI rats took longer to find the platform than sham rats. *, LI rats significantly different than sham rats; #, VEGF treated rats significantly different than VEH treated rats; *p* < 0.05. Sham+VEH, *n* = 10; Sham+VEGF, *n* = 10; LI + VEH, *n* = 11; LI + VEGF, *n* = 11. Error bar = SEM; water maze block = mean of every two trails.

In the open field, there was a main effect of treatment (*F*_1,38_ = 4.517, *p* < 0.05) on the percentage of time the female rats explored the middle, with VEGF females spending more time in the middle than VEH females (Figure 3A). There was also a main effect of injury (*F*_1,38_ = 4.922, *p* < 0.05) for total distance travelled, with female LI rats travelling less distance than shams (Figure 3B).

During water maze acquisition, there was also a significant injury by trial interaction (*F*_9,342_ = 3.396, *p* < 0.01) on search time with female LI rats spending more time than the female shams to find the platform on trials 6–8 (*p* < 0.05; Figure 3C). There were also main effects for injury (*F*_1,38_ = 7.238, *p* < 0.05) and for trial (*F*_9,342_ = 16.380, *p* < 0.001) on search time (Figure 3C). No significant findings were found on measures of direct and circle swims or swim speed (*p* > 0.05, data not shown).

During water maze reversal, there was a significant treatment by trial interaction (*F*_9,342_ = 2.950, *p* < 0.01), with female VEGF rats spending more time to find the platform on trials 4 and 8, when compared with VEH rats (*p* < 0.05; Figure 3D). There were also significant main effects for injury (*F*_1,38_ = 10.431, *p* < 0.01; LI rats > sham rats; Figure 3D) and for trial (*F*_9,342_ = 11.839, *p* < 0.001; Figure 3D). No significant findings were found on the measures of direct and circle swims or swim speed (*p* > 0.05, data not shown).

No significant findings were found in the EPM, beam task, or hot/cold plate for the female rats (data not shown, *p* > 0.05).

### VEGF treatment and mTBI alters hippocampal gene expression in both sexes

**Figure 4:**
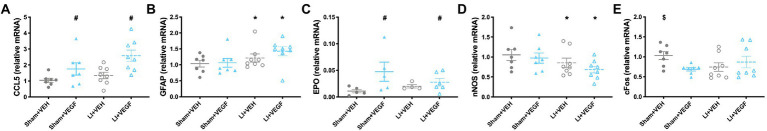
Gene expression in the hippocampus of male rats. Gene expressions of CCL5 **(A)** and EPO **(C)** were increased in VEGF rats compared with VEH rats. GFAP expression **(B)** increased and nNOS **(D)** decreased in LI rats compared with sham rats. The Sham+VEH group had significantly increased cFOS expression **(E)** compared with the Sham+VEGF group. *, LI rats significantly different than sham rats; #, VEGF treated rats significantly different than VEH treated rats; $, Sham+VEH rats significantly different than Sham+VEGF rats; *p* < 0.05. *n* = 7–8 per group. Error bar = SEM.

Genes related to vascular health, neuroinflammation, and hypoxia were assessed using multiplex qPCR. In males, there was a significant effect of treatment on the expression of *CCL5* (*F*_1,26_ = 11.509, *p* < 0.01; Figure 4A) and *EPO* (*F*_1,26_ = 4.605, *p* < 0.05; Figure 4C), both of which were increased in VEGF-treated rats compared with VEH-treated rats. There was also a main effect for injury on the expression of *GFAP* (*F*_1,26_ = 4.269, *p* < 0.05; Figure 4B) and *nNOS* (*F*_1,26_ = 4.290, *p* < 0.05; Figure 4D), with *GFAP* expression increased and *nNOS* decreased in LI rats compared with sham rats. There was an injury by treatment interaction for expression of *cFOS* (*F*_1,26_ = 4.594, *p* < 0.05; Figure 4E), with the Sham+VEH group having significantly increased *cFOS* expression compared with the Sham+VEGF group.

**Figure 5:**
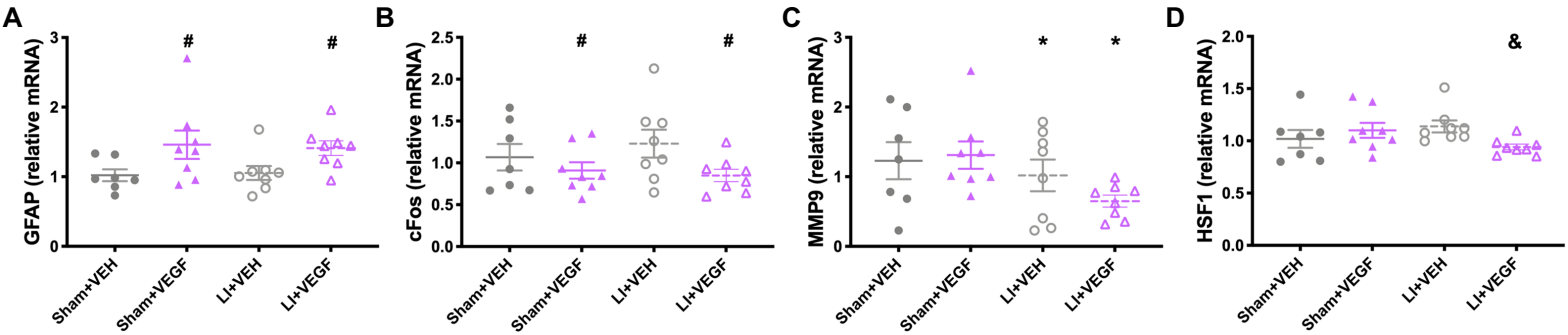
Gene expression in the hippocampus of female rats. VEGF rats had increased GFAP expression **(A)** and decreased cFOS expression **(B)** than VEH rats. **(C)** LI rats had less *MMP9* expression than sham rats. **(D)** LI + VEGF rats had decreased *HSF1* expression than the LI + VEH rats. *, LI rats significantly different than sham rats; #, VEGF treated rats significantly different than VEH treated rats; &, LI + VEGF significantly different than LI + VEH; *p* < 0.05. *n* = 7–8 per group, except for *EPO*, *n* = 4–6 per group due to undetectable levels in some samples. Error bar = SEM.

In females, there was a significant effect of treatment on the expression of *GFAP* (*F*_1,27_ = 8.686, *p* < 0.01; Figure 5A) and *cFOS* (*F*_1,27_ = 4.376, *p* < 0.05; Figure 5B), with VEGF-treated rats having increased *GFAP* expression and decreased *cFOS* expression compared with VEH-treated rats. There was also a main effect for injury on the expression of *MMP9* (*F*_1,27_ = 4.683 *p* < 0.05; Figure 5C), with LI rats having less than sham rats. There was an injury by treatment interaction for *HSF1* (*F*_1,27_ = 4.997, *p* < 0.05; Figure 5D), with the LI + VEGF group having decreased *HSF1* expression than the LI + VEH group.

No other statistically significant results were found for the other genes that were tested (data not shown).

## Discussion

This study examined the effects of recombinant VEGF-A treatment in male and female rats given a mTBI. The treatment was administered with ICV cannulation, beginning at injury onset and continuing for the 11-day duration of the experiment. In males, mTBI rats treated with VEGF-A took longer to find the platform in the water maze compared with all other groups, including mTBI rats treated with VEH, indicating that the VEGF-A treatment resulted in worse cognitive recovery after mTBI. In females, VEGF-A treatment resulted in worse water maze performance regardless of whether the rats were given a mTBI or sham injury. The female rats treated with VEGF-A also had decreased anxiety-like behavior in the open field, spending more time in the middle of the arena, regardless of injury. VEGF-A treatment was found to influence the expression of several genes in the hippocampus in both males (increased *CCL5* and *EPO*) and females (increased *GFAP* and decreased *cFOS*). There were other sex-specific findings in response to the mTBI, with only the mTBI males showing sensorimotor deficits on the beam, whilst only the mTBI females had decreased locomotion in the open field. In terms of mTBI changes in gene expression, mTBI increased *GFAP* and decreased *nNOS* in males whilst decreasing *MMP9* in female mTBI rats. Taken together, these findings indicate that the specific VEGF-A treatment protocol tested in this study failed to improve mTBI recovery in either male or female rats, with some evidence that it was actually detrimental.

Although these findings contradict our hypotheses and past findings (e.g., benefits of exercise in mTBI; benefits of VEGF in more severe preclinical TBI) (Feng et al., 2008; Lee and Agoston, 2010; Mychasiuk et al., 2016b; Kurowski et al., 2017; Leddy et al., 2019), there are several explanations that may account for these discrepancies. First, we began the VEGF-A treatment at the time of injury onset. VEGF-A, previously known as vascular permeability factor, is known to promote BBB permeability and may worsen cerebral edema after a brain insult (Croll et al., 2004; Koyama et al., 2007; Tado et al., 2014; Gao et al., 2015). Although mTBI is not typically associated with cerebral edema, it is possible that treatment with VEGF-A during the acute temporal window post-TBI when the brain is particularly vulnerable to edema may have resulted in worse behavioral outcomes. Of note, previous studies showing beneficial effects of exercise in mTBI do not start the intervention immediately post-injury; rather, the exercise treatment is typically delayed for days and even weeks after an mTBI (Mychasiuk et al., 2016b; Kurowski et al., 2017; Leddy et al., 2019). Therefore, timing of VEGF-A treatment or upregulation after mTBI could be critical in order to obtain beneficial effects and warrants further investigation. Although, it is also possible that the therapeutic effects of exercise in mTBI are due to other upregulated factors such as brain derived neurotrophic factor and not VEGF-A. With regards to the previous studies that found a neuroprotective effect for VEGF-A treatment or upregulation after more severe TBI (Lee and Agoston, 2010; Siddiq et al., 2012), it is important to note that other brain injury studies have reported conflicting results that are similar to ours and suggest that elevated VEGF-A may contribute to secondary injury mechanisms (e.g., neuroinflammation) and worse clinical symptoms (Croll et al., 2004; Gao et al., 2015).

Our gene expression findings provide some mechanistic insight into why the VEGF-A treatment had a negative impact on behavior measures. Neuroinflammation in the hippocampus has previously been associated with cognitive impairments after TBI (Aungst et al., 2014). We found that CCL5, a chemoattractant that recruits leukocytes and promote neuroinflammation (Gyoneva and Ransohoff, 2015), was elevated in the male rats treated with VEGF-A and may have contributed to poor outcomes in the mTBI + VEGF group. For example, in cerebral stroke CCL5 promotes neuroinflammation and cerebral microvascular dysfunction (Terao et al., 2008). We also found that VEGF-A treatment altered the expression of hypoxia and vascular related genes in male and female rats. EPO is upregulated in response to cellular hypoxia as it promotes erythropoiesis (Jha et al., 2018); thus our finding of upregulated *EPO* expression in VEGF-A treated males may suggest a hypoxic environment. cFOS can promote hippocampal neuronal survival after hypoxic insults (Ness et al., 2008) and is a marker of neuronal activity. Therefore, the decreased expression of *cFOS* in female rats treated with VEGF-A may help account for the cognitive deficits that were also observed in these rats.

There are several limitations to consider when interpreting the outcomes of this study. Any mechanistic speculation drawn from our gene expression analysis should be made with caution given the relatively low number of genes that were assessed. Future studies should incorporate a broader approach such as RNA-sequencing throughout a range of brain regions to establish a more comprehensive understanding of how VEGF-A treatment affects gene regulation in the brain after mTBI and how this relates to behavior. Transcriptional outcomes should also be complemented with analysis of protein expression. If neuroinflammation is a key mechanism, a more sophisticated analysis of how VEGF-A influences CNS immune cells such as microglia is warranted. Other mechanisms known to be influenced by VEGF-A such as neuronal proliferation and survival, as well as edema, should also be investigated. These analyses should include other post-injury timepoints, especially considering that the timing of our analysis at day 11 post-injury was likely less than ideal and may have missed the peak physiological response to both injury and treatment. The inclusion of a VEGF inhibitor in future studies may also help disentangle the mechanisms that underlie the detrimental and/or beneficial effects of VEGF-A treatment in different brain injury contexts (e.g., mild vs. severe).

In conclusion, the findings of this study do not support a treatment approach that involves immediate and continuous increase of cerebral VEGF-A after mTBI. With that said, our study was not without limitations and further studies into therapeutic window, dose, and underlying mechanisms are still requited to determine whether treatment with, or upregulation of, VEGF-A can be beneficial after mTBI and other forms of brain injury.

## Data availability statement

The raw data supporting the conclusions of this article will be made available by the authors, without undue reservation.

## Ethics statement

The animal study was reviewed and approved by the Alfred Medical Research and Education Precinct (AMREP) Animal Ethics Committee.

## Author contributions

SS and MS: conceptualization. MS, CW, RM, RB, and GY: animal models. MS, TB, and CW: behavioral testing. AV and TW: multiplex qPCR. MS, TB, and SM: data analysis. MS, TB, CW, RB, RM, GY, AV, TW, SM, and SS: manuscript writing and editing. All authors contributed to the article and approved the submitted version.

## Funding

The authors acknowledge funding support from the National Health and Medical Research Council of Australia (NHMRC) in the form of Project Grants and Career Development Fellowships to SS.

## Conflict of interest

The authors declare that the research was conducted in the absence of any commercial or financial relationships that could be construed as a potential conflict of interest.

## Publisher’s note

All claims expressed in this article are solely those of the authors and do not necessarily represent those of their affiliated organizations, or those of the publisher, the editors and the reviewers. Any product that may be evaluated in this article, or claim that may be made by its manufacturer, is not guaranteed or endorsed by the publisher.
